# Factors associated with transmission of influenza-like illness in a cohort of households containing multiple children

**DOI:** 10.1111/irv.12331

**Published:** 2015-08-04

**Authors:** Chelsea R Brown, James M McCaw, Emily J Fairmaid, Lorena E Brown, Karin Leder, Martha Sinclair, Jodie McVernon

**Affiliations:** a Centre for Epidemiology and Biostatistics, Melbourne School of Population and Global Health, The University of MelbourneParkville, Vic., Australia; b Modelling & Simulation Unit, Melbourne School of Population and Global Health, The University of Melbourne and Murdoch Children’s Research InstituteParkville, Vic., Australia; c Department of Microbiology and Immunology, The University of Melbourne at the Peter Doherty Institute for Infection and ImmunityParkville, Vic., Australia; d Infectious Disease Epidemiology Unit, School of Public Health and Preventive Medicine, Monash UniversityPrahran, Vic., Australia

**Keywords:** Household transmission, influenza, influenza-like illness, respiratory viruses, transmission

## Abstract

**Background:**

Household studies of influenza-like illness (ILI) afford opportunities to study determinants of respiratory virus transmission.

**Objectives:**

We examined predictors of ILI transmission within households containing at least two children.

**Methods:**

A prospective cohort study recorded ILI symptoms daily for 2712 adult and child participants during the 1998 influenza season in Victoria, Australia. Logistic and Poisson regressions were used to explore predictors of household transmission of ILI and the secondary household attack proportion (SHAP). A date of illness onset during the influenza season was used as a proxy indicator of ILI associated with influenza infection (as opposed to other aetiological causes).

**Results:**

A total of 1009 ILI episodes were reported by 781 of 2712 (29%) participants residing in 157 households. Transmission, defined as detection of ILI in one or more household members following identification of an index case, was observed in 206 of 705 (29%) household introductions. Transmission of ILI was significantly associated with the onset of ILI in the index case during the peak influenza season compared with the remainder of the observation period (37% versus 27%, odds ratio = 1·59, 95% CI 1·09, 2·31, *P* = 0·017). The SHAP was 0·12, higher if the index case was of secondary school age [incidence risk ratio (IRR) = 1·80, 95% CI 1·08, 2·98, *P* = 0·022].

**Conclusions:**

Risk of household transmission of ILI was increased during the peak influenza season, indicating an increased burden of disease during the period of influenza circulation. In this cohort, secondary-school-aged children and adults were important transmitters of ILI.

## Background

Influenza viruses are a major source of morbidity and mortality worldwide.^1^ The World Health Organization (WHO) estimates that influenza results in 3–5 million episodes of severe illness and one-quarter to half a million deaths each year throughout the world.^1^–^3^ Typical symptoms of acute influenza infection include but are not limited to fever, dry cough, headache, myalgia, lethargy, weakness, sore throat and coryza.^4^ The definition of influenza-like illness (ILI) for surveillance purposes usually comprises fever and at least one simultaneous respiratory symptom,^5^ although it is recognised that only a proportion of such illness will be attributable to influenza. Conversely, some true occurrences of influenza will not meet the case definition (due to, for example, lack of fever) or be entirely asymptomatic and not be recorded as an ILI.

Influenza and ILI affect people of all ages. Young children, the elderly, immunocompromised/immunosuppressed individuals, pregnant women and indigenous peoples are more vulnerable to severe influenza infection^6^,^7^. While the importance of different modes by which influenza infections are transmitted remains unclear,^4^ Hope-Simpson^8^ demonstrated that individuals with a household member with influenza were four times more likely to present with influenza than those without. Many subsequent studies have focused on households to explore onward transmission among adults and children living in close contact.

This study followed the experience of ILI within a cohort of households in Victoria, Australia, in 1998, including occurrence of secondary symptomatic cases to assess determinants of transmission. Temporal trends in the Victorian population’s experience of confirmed influenza infection were used to evaluate likely associations of influenza with onward spread of infection, compared with other circulating respiratory viruses.

## Method

### Sample

Families residing in 600 urban (Melbourne) Victorian households participated in an unrelated randomised controlled trial of measures to improve domestic water quality.^11^ Recruited households included at least four family members, with a minimum of two children under the age of 15 years. Daily health diaries were recorded for each of the family members, including the following symptoms of ILI: fever, chills and sweats and ‘cold’ (runny nose, sore throat and cough). Completed diaries were returned every 4 weeks, with the presence or absence of symptoms of ILI (and other data relevant to the water quality study) recorded daily for all family members. Missing data were minimal (see Results), and no adjustments were made.

### Study method

Utilising the prospective cohort study design, health diary data were assessed to explore the experience of ILI for all 2712 participants throughout the ‘influenza season’ of 1998 (defined to be 27th April to the 1st November 1998).

### The 1998 influenza season

**Figure 1:**
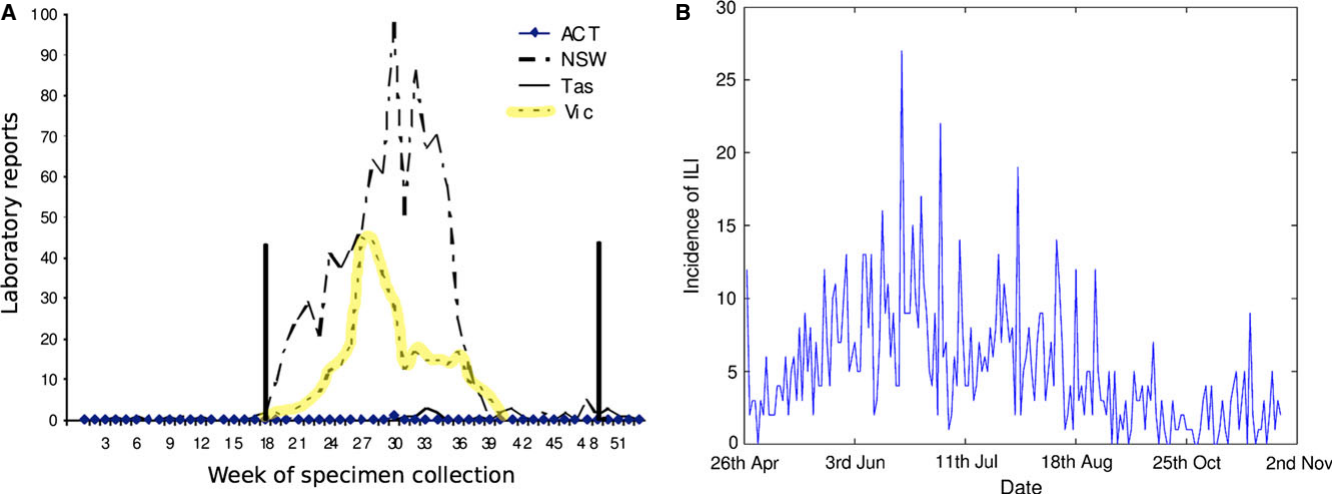
(A) Influenza A laboratory reports, by week, ACT, NSW, Tasmania and Victoria, 1998 (modified from Ref. 13). The Victorian influenza season is highlighted in yellow and the study period indicated by the vertical bars. (B) Incidence of influenza-like illness (ILI) in the cohort over the study period.

The Australian National Influenza Surveillance Scheme reported that 95% of the laboratory-confirmed cases of influenza in 1998 were identified as influenza A/Sydney/5/97 (H3N2). Surveillance data for 1998 (Figure1) indicate that there was a peak in the number of laboratory-confirmed influenza A (H3N2) cases in Victoria early in the influenza season between weeks 26 and 29 of 1998. These weeks correspond to the 4-week period spanning the 21st of June to the 18th of July in 1998. This 4-week period is referred to as the ‘peak period’ of the 1998 influenza season throughout this article. The A/Sydney/5/97 (H3N2) virus was antigenically similar to the emergent H3N2 variant that co-circulated with the earlier A/Wuhan/359/95 (H3N2) and influenza B strains in the 1997 season.^12^

The ILI syndromic definition (one systemic symptom: fever or chills/sweats, combined with at least one respiratory symptom: runny nose, sore throat or cough) was determined at the individual level via symptoms reported in the health diaries, and the full duration of the ‘symptomatic episode’ calculated for events within which these criteria were met. Within households, the temporal sequence of symptomatic illness was examined for the evidence of secondary infection (i.e. suspected transmission due to temporally related occurrence of ILI). A ‘household introduction’ was defined as the period of time during which one or more household member(s) (including the index case) experienced a symptomatic episode (i.e. a ‘household introduction’ includes the index case plus any temporally associated cases of ILI within the household). Following observation of four consecutive days in which all household members failed to meet the ILI case definition, we determined that the ‘household introduction’ had ceased. No household member was allowed to participate twice in a given ‘household introduction’. A gap of >4 days was defined as indicative of a new household introduction with a new index case. We chose this method to determine the end of one household introduction and the beginning of a new one, rather than the use of the serial interval for influenza, as we had no reliable estimates for the serial intervals for the full spectrum of possible aetiological causes of ILI. Note that with a focus on ILI, we chose to examine all household introductions in a given household, rather than only the first introduction during the study period.

### Additional testing (serology)

As part of the original study, three blood samples were collected from consenting adult participants at enrolment, the middle and the end of the 68-week period. For the purposes of this study, we selected enrolment samples collected between March 1997 and April 1998 to test for the evidence of immunity to the relevant circulating H3N2 strain (A/Sydney/5/97) prior to the 1998 season.^12^

Sera from blood samples were treated with receptor destroying enzyme (RDE) to remove non-specific inhibitors of haemagglutination. One volume of serum was added to 4·5 volumes of RDE (RDE II; Denka Seiken, Chuo-ku, Tokyo, Japan), and after overnight incubation at 37°C, an additional 4·5 volumes of 1·6% w/v sodium citrate were added and the mixture held at 56°C for 2 hours. Sera were then tested in a haemagglutination inhibition (HI) assay using the standard protocol.^14^ Briefly, RDE-treated sera were diluted in a doubling dilution series (25 μl) in 96-well round-bottomed microtitre trays and incubated for 30 minutes at room temperature with four haemagglutinating units of purified inactivated A/Sydney/5/97 (H3N2) virus in 25 μl. A 1% solution of chicken red blood cells (25 μl) was then added and the contents thoroughly mixed. After a further 30 minutes, the ability of specific antibodies in the sera to inhibit the virus-mediated haemagglutination of red cells was determined and the HI titre calculated. Evidence of previous exposure (through infection or vaccination, see Discussion) to the circulating strain or related variants was defined as a HI titre of ≥40.

### Statistical analyses

The primary outcome measure was onward transmission of symptomatic ILI to household members given a household introduction (yes/no). The exposure variables were household size, age group of index case, number of children in the household, introduction of symptomatic ILI into the household during the 4-week peak period of the influenza season and introduction during the remainder of the observation period. Univariate and multivariate logistic regression analyses were performed for these exposure variables.

The secondary outcome measure was the secondary household attack proportion (SHAP). The SHAP was calculated for each household introduction as the proportion of household contacts (i.e. the household size having excluded the index case) experiencing an individual symptomatic episode. Univariate and multivariate Poisson regression analyses were performed on the number of secondary household cases for household size, age group of index case, number of children in the household and introduction of ILI into the household during the influenza season, offset against the number of household contacts. Household clustering was accounted for through calculation of robust variance estimates.

For the purpose of a sensitivity analysis, the logistic regression analysis was also performed using an alternative peak period for the influenza season constrained to 2 weeks, from the 28th June to 11th July 1998 (see Figure1).

Transmission within the household and the SHAP were also examined with respect to the evidence for prior immunity to the circulating H3N2 strain in (one or more) household members, evidenced by a ‘pre-season’ HI titre at or above the putative protection threshold of 40.

Statistical analyses were conducted in stata ic versions 12 and 13 (StataCorp, College Station, TX, USA).

### Ethical approval

Ethical approval was sought and obtained from the University of Melbourne Human Research Ethics Committee to conduct this study using data and blood samples that were previously collected as part of the aforementioned water quality improvement study. Ethical approval was also sought and gained for the Use of Stored Human Tissue Samples from the Monash University Standing Committee on Ethics in Research involving Humans.

## Results

Approximately 43% of the study participants were adults, 11% were of secondary school age, 31% were primary school aged, and 14% were preschool aged. We obtained pre-season serology samples for 53% of all adult participants, 37% of which were seropositive (HI titre ≥40) to the circulating strain of influenza A (Table S1). Most households consisted of 4 (49%) or 5 (37%) people. The number of reporting household members ranged from 3 to 9, where we note that the two households with three reporting members both had four occupants but one person withdrew or did not participate.

Loss to follow-up was minimal with only 3·5% (99 participants of the originally recruited 2811) of participants withdrawing from the study before the end date used for this analysis. Of the 600 households recruited, 582 were retained in the study as of 1st November 1998, representing a loss of only 3% of all households. Missing data in the health diaries were minimal. In terms of participant-days, completeness percentages were 98·6%, 99·1% and 97·1% for ‘fever’, ‘chills and sweats’ and ‘cold’ respectively.

### Influenza-like illness infections

**Figure 2:**
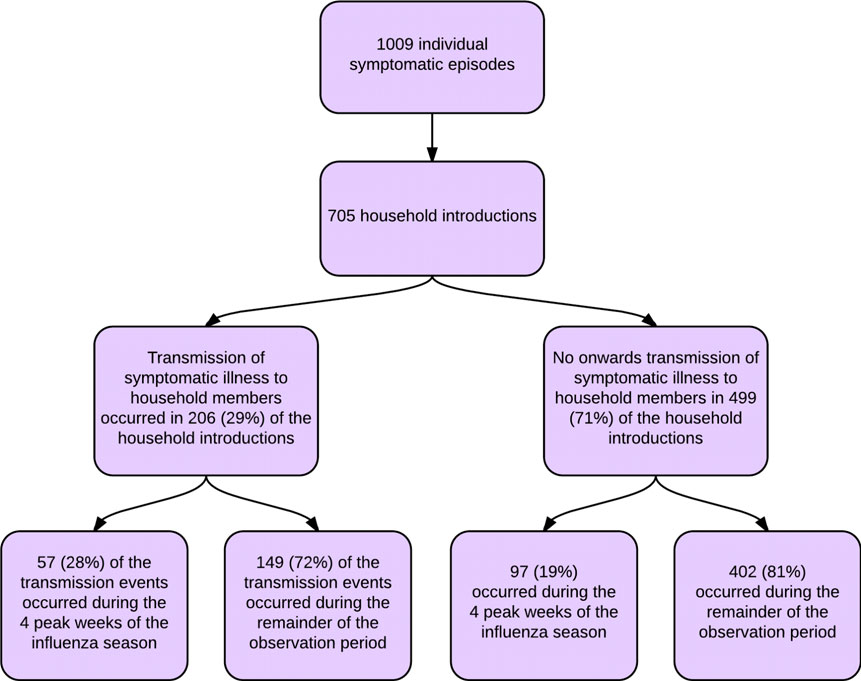
Flow diagram describing the individual symptomatic episodes and household introductions.

As summarised in Figure2, there were 1009 reported individual symptomatic episodes of ILI. An individual symptomatic episode was experienced at least once by 781 (28·8%) of the participants residing in 157 households. The majority (71·2%) of participants failed to meet the criteria of an individual symptomatic episode over the observation period and 7% of participants reported symptoms that met the case definition on two or more occasions.

The 1009 individual symptomatic episodes occurred within 705 household introductions (occurring in 360 unique households) involving one or more individual(s) (Table S2). While primary-school-aged children (349 from 855 participants) and adults (337 reports from 1162 participants) reported an ILI most frequently, preschool-aged children were the most likely to meet the case definition (214 reports from 386 participants).

Of the 614 adults for whom serology data were available, 36·8% had a pre-season HI titre of ≥40. Baseline seropositivity according to this threshold was not associated with a reduced chance of reporting an ILI episode (OR = 0·87, 95% CI 0·59, 1·29, *P *=* *0·51).

Onward transmission of ILI to household members occurred following 29·2% of the observed household ILI introductions. Almost one-quarter (23·5%) of the reported ILI episodes, and 28% of the episodes that resulted in onward transmission, occurred during the 4-week peak period of the influenza season.

### Factors associated with transmission of symptomatic illness within a household

Descriptive statistics and the results of the univariate and multivariate logistic regression models for the outcome of transmission of symptomatic illness within the household are displayed in Table1. Of the 154 household introductions of ILI that occurred during the 4-week influenza season, 57 (37·0%) resulted in onward transmission of symptomatic illness compared to 149 of 551 (27·0%) outside of the 4-week peak period. The increased odds [1·59 (1·09, 2·31), *P *=* *0·017] of transmission of ILI during the influenza season indicate an increased burden of ILI during the influenza season, perhaps due to an increased transmissibility of the influenza virus compared to other aetiological causes of ILI, although alternative explanations are possible (see Discussion). Baseline seropositivity was not associated with any change in the risk of onward transmission of ILI, over the entire study period or during the 4-week influenza season (data not shown).

**Table 1:** Predictors of transmission. Transmission of symptomatic illness occurred in 206 of the 705 reported household introductions

Explanatory variable		Transmission % of number of reported household introductions*	Univariate logistic regression		Multivariate logistic regression	
			OR (95% CI)	*P*	OR (95% CI)	*P*
Household size	4	86 of 328 (26·2%)	Reference	–	1·00	–
	5	87 of 260 (33·5%)	1·42 (0·99–2·02)	0·056	1·46 (1·01–2·09)	0·042
	6+	33 of 117 (28·2%)	1·11 (0·69–1·77)	0·677	1·10 (0·68–1·78)	0·688
Age group of index case	Preschool 0 to <5 years	31 of 143 (21·7%)	Reference	–	1·00	–
	Primary school 5 to <12 years	71 of 239 (29·7%)	1·53 (0·94–2·48)	0·087	1·61 (0·99–2·63)	0·057
	Secondary school 12 to <19 years	26 of 77 (33·8%)	1·84 (0·99–3·42)	0·053	1·90 (1·02–3·53)	0·044
	Adult 19 years and over	78 of 246 (31·7%)	1·68 (1·04–2·71)	0·035	1·77 (1·09–2·88)	0·022
Number of children <19 years in house	2 or less	89 of 330 (27·0%)	Reference	–	Not included	–
	3	86 of 264 (32·6%)	1·31 (0·92–1·87)	0·137	Not included	–
	4+	31 of 111 (27·9%)	1·05 (0·65–1·70)	0·844	Not included	–
Onset of reported household introduction (cf. remainder of observation period)	Remainder of observation period outside 4-week peak period**	149 of 551 (27·0%)	Reference	–	1·00	–
	4-week peak period of influenza season**	57 of 154 (37·0%)	1·59 (1·09–2·31)	0·017	1·60 (1·09–2·34)	0·016
	Remainder of observation period outside 2-week peak period***	176 of 632 (27·9%)	1·00	–	Not included	–
	2-week peak period of influenza season***	30 of 73 (41·1%)	1·81 (1·10–2·97)	0·020	Not included	–

CI, confidence interval; OR, odds ratio.

* A reported household introduction involved one or more household members having an individual symptomatic episode.

** Four-week peak period of laboratory-reported influenza in 1998 between June 21st and July 18th.

*** Two-week peak period of laboratory-reported influenza in 1998 between June 28th and July 11th.

The strongest predictor of transmission of symptomatic ILI in the multivariate model was the age group of the index case, with adults (OR = 1·77, 95% CI 1·09, 2·88, *P *=* *0·022) and secondary school students (OR = 1·90, 95% CI 1·02, 3·53, *P *=* *0·04) posing a higher risk to family members relative to preschool students, in the analysis performed on the full data set. Onset of illness during the peak influenza season remained influential in this model (OR = 1·60, 95% CI 1·09, 2·34, *P *=* *0·016).

### Factors associated with the number of secondary illness reports given a household introduction

Within the 705 reported household introductions, there were a total of 304 secondary cases among 2639 household contacts (SHAP = 0·115). The SHAP was greatest for household introductions with a secondary-school-aged index case (41 secondary cases reported among 296 household contacts (SHAP = 0·139), see Table2).

**Table 2:** Predictors of the number of secondary cases in the household, offset against the number of susceptible household contacts (secondary cases occurred in 206 of the 705 reported household introductions)

Explanatory variable		SHAP for group (no. of reported household introductions*)	Univariate Poisson regression		Multivariate Poisson regression	
			IRR (95% CI)	*P*	IRR (95% CI)	*P*
Household size	4	Reference	1·00	–	1·00	–
	5	0·125 (260)	1·04 (0·78–1·39)	0·775	1·59 (0·82–3·05)	0·168
	6+	0·091 (117)	0·76 (0·50–1·14)	0·193	1·00 (0·42–2·34)	0·992
Age group of index case	Preschool 0 to <5 years	Reference	1·00	–	1·00	–
	Primary school 5 to <12 years	0·116 (239)	1·51 (1·00–2·27)	0·048	1·56 (1·04–2·35)	0·031
	Secondary school 12 to <19 years	0·139 (77)	1·80 (1·09–2·98)	0·002	1·86 (1·13–3·07)	0·015
	Adult 19 years and over	0·129 (246)	1·68 (1·12–2·51)	0·012	1·71 (1·14–2·56)	0·010
Number of children <19 years in house	2 or less	Reference	1·00	–	1·00	–
	3	0·122 (264)	1·00 (0·75–1·32)	0·973	0·66 (0·34–1·26)	0·208
	4+	0·091 (111)	0·74 (0·49–1·14)	0·170	0·74 (0·30–1·80)	0·509
Onset of reported household introduction*	Remainder of observation period outside 4-week peak period**	Reference	1·00	–	1·00	–
	4-week peak period** of influenza season	0·137 (154)	1·26 (0·94–1·68)	0·119	1·26 (0·94–1·67)	0·117

CI, robust confidence interval; SHAP, secondary household attack proportion; IRR, incidence risk ratio.

* A reported household introduction involved one or more household members having an individual symptomatic episode.

** Four-week peak period of laboratory-reported influenza in 1998 between June 21st and July.

Commensurate with the logistic analysis, but not statistically significant, the SHAP tended to be larger for household introductions that occurred during the 4-week peak period of the influenza season compared to the remainder of the observation period: 78 secondary cases of ILI reported among 568 susceptible household contacts (SHAP = 0·137), compared with 226 secondary cases of ILI among 2071 susceptible household contacts (SHAP = 0·109) during the remainder of the observation period [incidence rate ratio (IRR) = 1·26, 95% CI 0·94, 1·68, *P *=* *0·119]. Table2 summarises the results of the SHAP analyses and the univariate and multivariate Poisson regression models. Again, baseline seropositivity was not associated with any change in the SHAP, over the entire study period or during the 4-week peak period of influenza circulation (data not shown).

There was a large and statistically significant effect of age group of the index case in the analysis performed on the full data set. The strongest predictors of the number of secondary cases of ILI were a secondary-school-aged index case (IRR = 1·86, 95% CI 1·13, 3·07, *P *=* *0·015) and an adult introducer (IRR = 1·71, 95% CI 1·14, 2·56, *P *=* *0·010), consistent with the findings from the multivariate logistic regression model for transmission.

## Discussion

An individual symptomatic episode occurring during the peak period of the influenza season was more likely to result in onward household transmission to household contacts (OR = 1·60, 95% CI 1·09, 2·34, *P *=* *0·016). Similarly a positive association, although not statistically significant, was found during the peak period for the number of secondary cases reported in a household introduction (IRR = 1·26, 95% CI 0·94, 1·67, *P *=* *0·117). These findings provide the evidence for an increased burden of ILI during the peak period of influenza transmission, consistent with previous reports of the heightened infectiousness of influenza compared with other respiratory viruses.^10^ However, in the absence of virological testing to determine the aetiological cause of ILI, other explanations for this increased burden are possible. Driven by temporal changes in environmental conditions (e.g. temperature, humidity), clinical expression of infection may change during the study period, or viruses may be more readily transmitted. Of note, our ILI definition requires the presence of ‘fever’ that may preclude identification of milder illness. Alternatively, during periods of increased ILI incidence, the rate of introduction to the household would also be expected to increase, potentially explaining the increased level of transmission.

Age group of the index case was a strong predictor of transmission of symptomatic illness in a household introduction. Of note, introductions by secondary-school-aged children were associated with a higher number of secondary cases relative to introductions from preschool-aged children (IRR = 1·86, 95% CI 1·13, 3·07, *P *=* *0·015). These results signal an elevated burden of ILI within the household based on age group of the index case. We note that multiple explanations for this observation are available, including an increased transmissibility of the (unknown) virus from these age groups, or age dependencies in the clinical manifestation of infection.

The present prospective cohort study observed a sample of households containing at least two children over the Australian influenza season of 1998, and so was enhanced for infection opportunity. Detailed information on symptomatic illness was collected for all participating household members. Serological data from a subset of adults provided limited information on possible previous household immunity to the strain of H3N2 circulating during 1998. No evidence was found that pre-season immunity was associated with changes in individual- or household-level experience of ILI. We note that although vaccine history was not available, low levels of influenza vaccine receipt among healthy Australian adults and children in 1998^15^ make it unlikely that observations of baseline seropositivity in this cohort were due to immunisation. The ‘gold standard’ for the study of influenza transmission is a cohort study.^16^ Given that the main source of potential bias in a cohort study is loss to follow-up,^17^ the present study provided a rich and high-quality data set in which to examine factors associated with the burden and transmission of ILI. As with all studies that rely on any form of syndromic criteria for the identification of household introductions, we cannot directly account for the fact that infection may have been introduced into the household by those who were either asymptomatic or had an illness that did not meet the ILI definition. However, this is of minimal consequence given our emphasis on the burden and transmission of ILI, rather than transmission characteristics of the causative pathogen(s).

Households provide for the close interaction among members of varying age groups within a confined space, providing excellent opportunity for the examination of transmission of influenza and other respiratory viruses.^9^ A recent systematic review and meta-analysis^9^ included 27 studies on household transmission of 2009 pandemic Influenza A. The review concluded that the varied methods for index case ascertainment, different recruitment strategies and disparate study designs were an important source of heterogeneity.^9^ The main recruitment method employed in 23 of the 27 studies was case ascertainment, contrasting with our ‘gold standard’ cohort approach. Using case ascertainment as a recruitment method may result in a skewed representation of ILI/influenza cases and arguably an upwardly biased estimate for transmissibility, as only those cases severe enough to seek medical care are captured within the surveillance data and subsequently recruited to participate.

The majority of household introductions in this study were attributed to children of primary school age and adults. This profile was consistent with the observed epidemiology of influenza in 1998, during which all age groups were broadly represented among general practice ILI consultations, peaking in the middle years, validating the cohort’s ability to capture community experience.^13^ In contrast, notifications in that same year peaked among 0–4 year olds.^13^ Onward transmission of influenza was more likely when the index case was older than 5 years of age, with the greatest odds observed among secondary-school-aged children. This observation, contrasting with earlier findings of McCaw *et al*.^10^ of an enhanced role for younger children, may in part be attributed to the study design which required households to contain at least two children under 15 years of age. While difficult to assess, older school-aged index cases would be anticipated to have at least one younger sibling, who by virtue of age would likely be susceptible to acquisition of infection in the household setting. Indeed, preschool-aged children showed the highest rates of ILI within the cohort (214 reports from 386 participants). Further study of the role of age of household members is warranted.

## Conclusions

Our findings are a reminder of the role that school children of all ages play in the introduction and onward transmission of infection to family members. These observations endorse the recent UK recommendation to immunise all children from 2 to 16 years of age with live attenuated influenza vaccine. Preliminary evaluation of this program, delivered up to 11 years of age during the 2013/2014 influenza season, indicates both direct and indirect protective effects.^18^ It remains to be seen whether additional age and population coverage will accentuate such impacts, given the suggested importance of older children to infection spread as identified in our study.

## Supporting Information

**Table S1.** Summary statistics by age group in a prospective cohort study of respiratory illness in 600 households of Melbourne. **Table S2.** Total number of ILI infections, ILI infections during four peak weeks of influenza season, household introductions and onwards transmission by age group in 600 households of Melbourne.
