# Supplementary material for: Factors associated with transmission of influenza-like illness in a cohort of households containing multiple children
Source: Influenza Other Respir Viruses. 2015 Aug 4;9(5):247–54. doi: 10.1111/irv.12331 (PMC4548994; doi:10.1111/irv.12331)
Supplement: Supplementary file 1 [file irv0009-0247-sd1.docx]

# Supplementary Tables

**Table S1. Summary Statistics by age group in a prospective cohort study of respiratory illness in 600 households of Melbourne.**

| Variable | Mean [SD], range; or N (%) | | | | |
| --- | --- | --- | --- | --- | --- |
|  | Pre-school age  <5 years  n = 386 (14.2%) | Primary school age  5-<12 years  n = 855 (31.5%) | Secondary school age  12-<19 years  n = 309 (11.4%) | Adult 19+ years  n = 1162 (42.9%) | Total  N = 2712 (100%) |
| Female | 172 (44.6%) | 397 (46.4%) | 162 (52.4%) | 589 (50.7%) | 1320 (48.7%) |
| Age, years | 3.33 [1.1],  0.74-4.99 | 8.25 [1.99],  5.01-11.99 | 14.16 [1.59],  12.01-18.77 | 38.10 [5.27],  19.27-58.04 | 21.0 [15.5],  0.74 – 58.04 |
| Pre-season positive titre ^†^ | - | - | - | 226/614 (36.8%) | 226/614 (36.8%) |

N = 2712; SD – Standard deviation; † - A positive-titre was determined by a Haemagglutination Inhibition titre for influenza A virus of >= 40, collected in a subset of 614 adults.

**Table S2. Total number of ILI infections, ILI infections during four peak weeks of influenza season, household introductions and onwards transmission by age group in 600 households of Melbourne.**

| Variable | Proportion of events (# of reported events) | | | | |
| --- | --- | --- | --- | --- | --- |
|  | Pre-school age  <5years | Primary  school age  5-<12years | Secondary school age  12-<19 years | Adult  19+ years | Total |
| Reported ILI individual infections | (214) | (349) | (109) | (337) | (1009) |
| Household introducer | 66.8% (143) | 68.5% (239) | 70.6% (77) | 73% (246) | 69.9%  (705/1009) |
| Transmission to household members | 21.7% (31) | 29.7% (71) | 33.8% (26) | 31.7% (78) | 29.2% (206/705) |
| Reported ILI infection during  4 week peak period^§^ of  ‘flu season’ | 24.8% (53) | 22.6% (79) | 19.3% (21) | 24.9% (84) | 23.5% (237/1009) |

N = 2712; ILI – influenza-like illness, § - four week peak period of laboratory reported influenza in 1998 between June 21^st^ and July 18^th^.
